# Mapping PP1c and Its Inhibitor 2 Interactomes Reveals Conserved and Specific Networks in Asexual and Sexual Stages of *Plasmodium*

**DOI:** 10.3390/ijms23031069

**Published:** 2022-01-19

**Authors:** Caroline De Witte, El Moukhtar Aliouat, Cerina Chhuon, Ida Chiara Guerrera, Christine Pierrot, Jamal Khalife

**Affiliations:** 1 Univ. Lille, CNRS, Inserm, CHU Lille, Institut Pasteur de Lille, U1019—UMR 9017—CIIL—Centre d’Infection et d’Immunité de Lille, 59000 Lille, France; caroline.de-witte@pasteur-lille.fr (C.D.W.); el-moukhtar.aliouat@univ-lille.fr (E.M.A.); 2 Proteomics Platform Necker (PPN), Université de Paris, Structure Fédérative de Recherche Necker (SFR Necker, INSERM US24/CNRS UAR3633), 75015 Paris, France; cerina.chhuon@inserm.fr (C.C.); chiara.guerrera@inserm.fr (I.C.G.)

**Keywords:** protein phosphatase 1, inhibitor 2, *Plasmodium*, interactome, dephosphorylation, PP1 partners

## Abstract

Malaria parasites require multiple phosphorylation and dephosphorylation steps to drive signaling pathways for proper differentiation and transformation. Several protein phosphatases, including protein phosphatase 1 (PP1), one of the main dephosphorylation enzymes, have been shown to be indispensable for the *Plasmodium* life cycle. The catalytic subunit of PP1 (PP1c) participates in cellular processes via dynamic interactions with a vast number of binding partners that contribute to its diversity of action. In this study, we used *Plasmodium berghei* transgenic parasite strains stably expressing PP1c or its inhibitor 2 (I2) tagged with mCherry, combined with the mCherry affinity pulldown of proteins from asexual and sexual stages, followed by mass spectrometry analyses. Mapped proteins were used to identify interactomes and to cluster functionally related proteins. Our findings confirm previously known physical interactions of PP1c and reveal enrichment of common biological processes linked to cellular component assembly in both schizonts and gametocytes to biosynthetic processes/translation in schizonts and to protein transport exclusively in gametocytes. Further, our analysis of PP1c and I2 interactomes revealed that nuclear export mediator factor and peptidyl-prolyl cis-trans isomerase, suggested to be essential in *P. falciparum*, could be potential targets of the complex PP1c/I2 in both asexual and sexual stages. Our study emphasizes the adaptability of *Plasmodium* PP1 and provides a fundamental study of the protein interaction landscapes involved in a myriad of events in *Plasmodium*, suggesting why it is crucial to the parasite and a source for alternative therapeutic strategies.

## 1. Introduction

Malaria is a parasitic disease of major public health importance caused mainly by *Plasmodium falciparum*. Its global control is still under serious threat due to the intensive use of the available treatments, participating at least in part of the emergence of multidrug resistance that contributes to a higher risk of transmission, morbidity, and mortality [1]. Consequently, the search for new targets and drugs is imperative.

In this context, one strategy is to decipher orthologues of proteins described to play essential roles in other eukaryotes in order to specifically target them in the malaria parasite. Among these proteins, due to the fact of their pleiotropic roles in the dynamic regulation of protein function and cellular distribution, enzymes involved in dephosphorylation processes and, in particular protein phosphatase 1 (PP1), are of interest. PP1 is among the most active drivers of protein dephosphorylation [2]. In humans, there are three PP1 catalytic (PP1c) isoforms expressed by three distinct genes [3]; however, *Plasmodium* spp. possess only one PP1c. Initial experiments using natural phosphatase inhibitors have shown that okadaic acid was able to drastically inhibit *P. falciparum* growth in vitro [4,5]. On the basis of the high IC_50_ and IC_90_ values observed with okadaic acid in these studies, it was suggested that the significant decrease in growth could mainly be due to PP1. More recently, compelling evidence from genetic studies clearly demonstrated the implication of PP1c not only in the *Plasmodium* division but also in egress of the blood stage parasites [6]. The PP1 catalytic subunit itself could therefore be considered a promising target for the development of innovative and effective drugs against malaria. However, this could be difficult to achieve, as PP1c is highly conserved in eukaryotes.

At the molecular level, it is well known that PP1c forms and functions as a holoenzyme with a vast network of binding partners. Hence, it is reasonable to expect that acting on PP1c interactions or interactors in *Plasmodium* may lead to the development of selective drugs. In yeast and mammals, PP1c has been shown to function almost exclusively in complexes [7,8,9,10] with diverse regulators/partners to dephosphorylate a plethora of proteins involved in many cellular processes including glycogen metabolism, transcription or mitosis [2]. So far, hundreds of partners have been reported, and many of them have been shown to be involved in the coordination of PP1c activities. These binding partners can be PP1c substrates, recruit additional substrates, transport PP1c, and/or regulate the phosphatase activity. A hallmark of most PP1c regulators is that they exhibit an RVxF sequence as a primary binding motif [7]. At the cellular level, converging studies clearly reported essential functions of some conserved regulators, such as sds22, inhibitors 2 (I2) and 3 (I3), suggesting that the specificity of action of PP1c may originate from the partner rather than from catalytic subunit itself [2].

In *Plasmodium*, we used different approaches combining the study of known conserved regulators, the use of the yeast 2-hybrid approach in which PfPP1c was the bait as well as immunoprecipitated (IP) complexes of blood parasites using HA-tagged PP1c followed by mass spectrometry analyses (MS) to examine the PP1c interactome [11]. This allowed the confirmation of the presence of the conserved sds22 (designated LRR1 in *Plasmodium*) and I2 and I3 partners. Their crucial role was subsequently demonstrated as disrupting their interaction with PfPP1c, induced the death of blood stage parasites [12,13]. In addition, we reported a high number of specific PP1c *Plasmodium* partners including GEXP15, whose deletion drastically affected the growth of blood stage parasites and completely blocked the development of oocysts and sporozoites in the mosquito [14]. While these early approaches started to pinpoint the *Plasmodium* PP1c interactome, they reported a static picture of the interactome, since they focused on asexual schizonts. Hence, it was crucial to confirm and to further extend the PP1c network mapping to other parasite stages. This was reinforced by a recent study in which PP1c was shown to be highly expressed in gametocyte sexual stages and to regulate chromosome segregation during mitotic and meiotic division of sexual stages [15]. Based on the idea that PP1c interactive protein partners and their pathways could co-evolve and/or need to adapt, we therefore sought to characterize the intricate PP1c interaction networks at two parasite stages to increase understanding of how this enzyme could execute its multiple functions. For this purpose, we used the IP/MS approach to allow active network mapping of direct interactions and indirect associations. We first examined *Plasmodium berghei* schizonts and gametocytes expressing tagged mCherry–PP1c to identify PP1c protein complexes. Further, reciprocal experiments with a direct partner of PP1c, I2 were performed to confirm and to uncover potential PP1c–I2 pathways, using the same strain of *P. berghei* that we generated during this study to express mCherry–PbI2. We present, here, the interactomes of PbPP1c and PbI2, showing common and specific interactors depending on the *Plasmodium* parasite stage. The network analysis of the PP1c-associated proteins revealed dynamic signaling networks responsive to parasite stage development, ranging from cellular component assembly, translation, and protein transport. These data support the capacity of a functional adaptation of PP1 to contribute to diverse and essential biological processes and provide a basis for studies targeting PP1 holoenzymes in *Plasmodium*.

## 2. Results and Discussion

### 2.1. Transgenic P. berghei Lines Expressing Tagged Baits

The aim of this study was to identify PP1c partners in order to better unravel its biological roles and functions in *Plasmodium* asexual and sexual stages. To address the analysis of its interactions and its formed complexes, we conducted an IP-MS based study using tagged proteins in *P. berghei*. Our selected protein baits were PP1c and its I2 regulatory subunit. The generation of a *P. berghei* line expressing mCherry-tagged PP1c has previously been reported [14]. As for PbI2, a parasite line expressing mCherry-tagged I2 from its endogenous locus was generated in the same strain used for PbPP1c. Genotyping analysis showed the correct integration of the tagged PbI2 and the expression of mCherry–PbI2 was checked by immunoblot using anti-RFP antibody (Supplementary Materials Figure S1). It is important to point out that the *P. berghei* ANKA strain used throughout this study produced gametocytes with expected ookinete conversion rates (data not shown).

**Figure 1:**
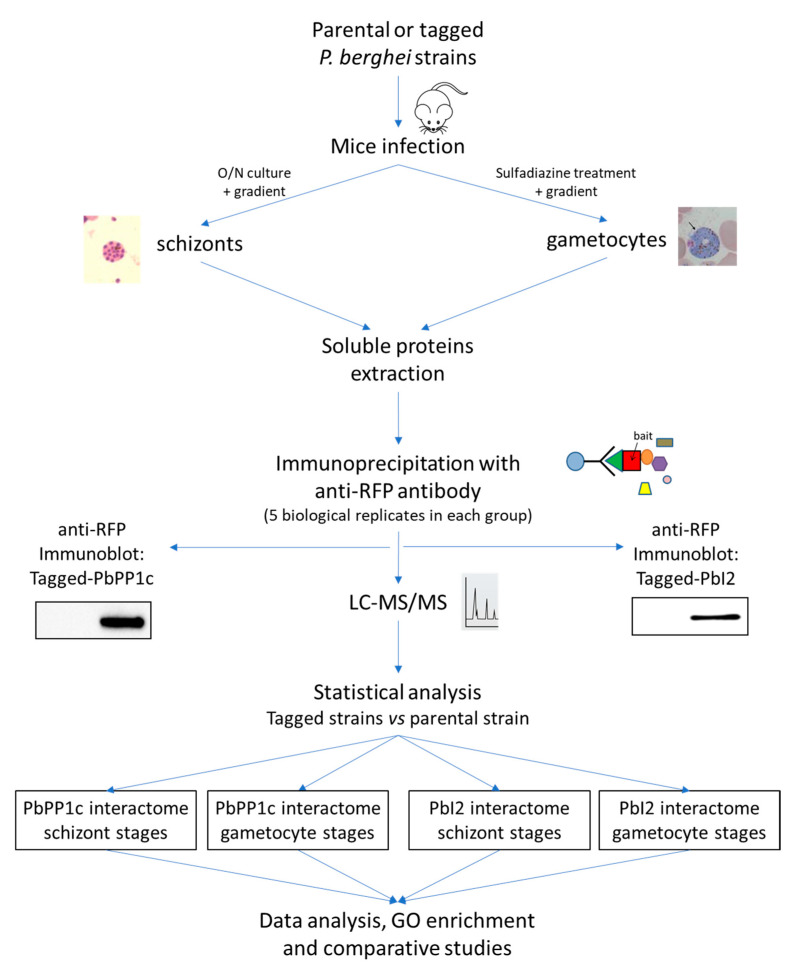
Workflow and study overview. Schizont and gametocyte stages were prepared from PbWT GFP, PbPP1c–mCherry, and PbI2–mCherry parasites. Immunoprecipitation was performed with anti-RFP antibody on 5 different biological replicates of schizonts and gametocytes extracts prepared from each parasite strain (PbWT, PbPP1c–mCherry, and PbI2–mCherry). The presence of mCherry-tagged PbPP1c and PbI2 was checked in the immunoprecipitated fractions (control wild-type vs. tagged proteins) before MS. Proteomic and statistical analyses (detailed in Section 3) allowed for the identification of the interactome of PbPP1c and PbI2 in schizont and gametocyte stages that were further analyzed for GO enrichment, STRING networks, and comparative analyses.

For proteomic experiments, a schematic illustration of the methodology used is shown in Figure 1. Schizonts and gametocytes were purified, and IP experiments were performed with proteins extracted from five independent biological replicates using beads coupled to anti-RFP antibody (see Section 3 for details). Prior to MS proteomic analysis, the presence of tagged baits in each IP was checked by immunoblot (illustrated in Figure 1). As control experiments, we performed IP on proteins extracted from the same parasite stages of the parental wild-type strain. In all experiments, the eluted PbPP1c and PbI2-interacting complexes were analyzed by LC-MS/MS.

### 2.2. Identification of PbPP1c-Interacting Proteins in Schizonts and Gametocytes

**Figure 2:**
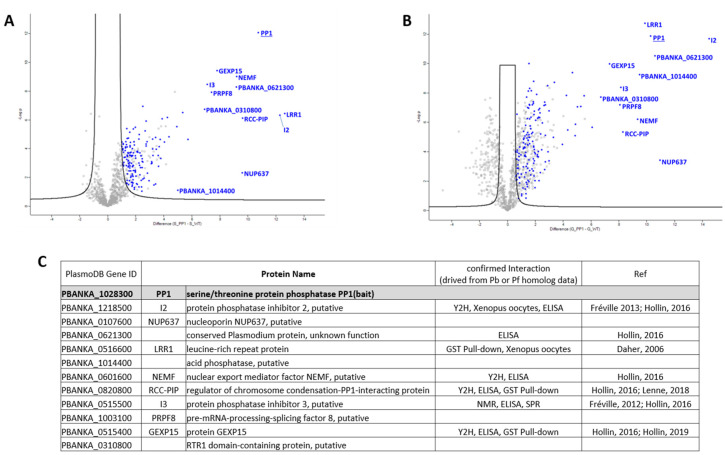
PbPP1c interactome analysis. (**A**,**B**) Volcano plot representations of the outcome of the PbPP1c interactome study carried out in schizonts (**A**) and in gametocytes (**B**). Highlighted in blue are the common interacting proteins (CIPs) in PbPP1c interactomes in schizont and gametocyte stages. The top PbPP1c-interacting proteins are annotated (see **C**). (**C**) List of top PbPP1c-interacting partners. Proteins were ranked according to their Student’s *t*-test difference PbPP1c–PbWT in schizont and gametocyte stages, and the top 10 proteins at each stage are listed. Protein description was updated using PlasmoDB relapse 54 (September 2021). Confirmed interaction data from previous works are in the two last columns [11,12,14,16–18]. Y2H and NMR stand for yeast 2-hybrid and nuclear magnetic resonance, respectively.

The digested samples were directly analyzed by LC-MS/MS using five biological replicates to identify PbPP1c-associated proteins. After statistical analysis, the data revealed a total of 489 proteins including the mCherry–PbPP1c bait protein. Among them, 157 proteins were present in both schizont and gametocyte extracts and considered as common interacting proteins (CIPs) (Supplementary Materials Table S1, sheets 2–4 in pink; Figure 2). In addition, 68 proteins were detected as specific interacting proteins in schizonts (SIPS) (Supplementary Materials Table S1, sheet 2 in green), and 263 proteins were found as specific interacting proteins in gametocytes (SIPGs) (Supplementary Materials Table S1, sheet 3 in blue). Among the CIP, it is important to point out that LRR1, I2, I3, GEXP15, and RCC–PIP proteins as well as NEMF and PBANKA_0621300 proteins, which we previously reported as direct interactors of *Plasmodium* PP1c, were found among the top interacting proteins (Figure 2, annotated in panels (A) and (B) and listed in panel (C)) [11,12,14,16,17,18]. This observation supports the robustness of the interactome data as well as a validation of the IP/MS experiments. Next, we analyzed all potential PbPP1c protein complexes (using the Protein Motif Pattern tool of PlasmoDB) for the presence of the highly conserved RVxF docking motif [19], known to be present in >80% of PP1c direct regulators [7]. Of the 488 proteins, 296 interacting proteins exhibited at least one consensus RVxF binding motif (Supplementary Materials Table S1). These motifs are enriched when compared to the overall presence of the RVxF motif in *P. berghei* proteins (fold 1.17; hypergeometric *p*-value = 3.17 × 10^5^, https://systems.crump.ucla.edu/hypergeometric/index.php, accessed on 16 August 2021). This suggests that the presence of the RVxF motif could contribute to the molecular basis of interaction with PP1c. These motifs need to be further investigated and confirmed as structural and functional motifs.

Following the PbPP1c immunoprecipitation, it should be noted that 21% of the CIPs (34/157) and 45% of the SIPS (31/68) were found to be ribosomal subunits or ribosome-associated proteins (Supplementary Materials Table S1), suggesting that PbPP1c brings down a large part of the ribosome complex. It is very unlikely that these interactions could be considered as non-specific. In support for this, the MS of IP extracts of WT parasites and of the mCherry–PbI2 parasites generated in the same strain in which the mCherry–PbPP1c line was obtained did not allow for the detection of these proteins (see below).

In addition, it is important to bear in mind that previous studies support the selectivity of PP1c binding to ribosome complexes in mammalian cells [20,21,22]. Further, by manual curation, we found that 19 ribosome-associated proteins detected in the interactome exhibited at least one RVxF binding motif, supporting their potential direct interactions with PbPP1c (Supplementary Materials Table S1). Further, the detection in schizonts of Nop52, known to contribute to ribosome production and to interact with PP1 through its RVxF binding motif [23], among the main interactors in our IP-MS of mCherry–PbPP1c, argues in favor of selective PP1c–ribosome complex interactions. Of note, *Plasmodium* Nop52 contains two potential RVxF binding motifs to PP1c. As for the presence of ribosomal protein complexes in CIP and also in SIPS, this could be indicative of a distinct role of PP1 at different levels during the biogenesis of ribosomes in schizonts by regulating their phosphorylation status. Indeed, among the protein complexes in CIP, we found 5 subunits corresponding to initiation factor 3, supportive of a potential regulation of the initiation of translation. Along the same line, our set of data with the total proteins interacting with PP1c showed that they are present in the cytoplasm and the nucleus. This is consistent with the idea that *Plasmodium* PP1c has the capacity to establish a high number of separate protein complexes in both compartments. However, additional investigations are still required to capture transient and fast exchanging PP1c interacting proteins by the use, for instance, of cross-linking methods and to examine which partner functionally influences the other.

### 2.3. Gene Ontology Enrichment Analyses of PbPP1c-Interacting Proteins

In order to gain some insight into potential pathways in which PP1c could be involved and to avoid bias in their identification, GO enrichment pathways including all detected proteins in each parasite stage were explored using PlasmoDB software (release 51, March 2021). We analyzed enriched GO terms/pathways of computed and curated biological processes. In the case of schizonts, 37 pathways were found to be enriched, including 5 with a strong fold enrichment (>5) involved in spliceosomal SnRNP assembly, formation of cytoplasmic translation initiation complex, cytoplasmic translation, ribonucleoprotein complex assembly, and subunit organization (Supplementary Materials Table S2). Closer examination of GO-enriched terms revealed that a large list of genes had multiple annotations and, in some cases, GO pathways included shared or the same set of genes, leading to overlap and duplication. Consequently, and in the search for the most informative data, full GO terms were reduced using the GO slimmer tool (PlasmoDB release 51) to obtain Slim annotations that were further inspected to reassign, when appropriate, to GO parent terms (Table 1). However, general GO terms were excluded (such as biosynthetic process and metabolic process) while branch information was retained to ensure that the enriched family of each function was present in the data. The results showed that, in schizont stages, PbPP1c was predominantly associated with “cellular component assembly” (20 proteins), encompassing “protein-containing complex assembly” (18 proteins), “ribonucleoprotein assembly” (13 proteins), and in “translation” complex (57 proteins) (Table 1).

**Figure 3:**
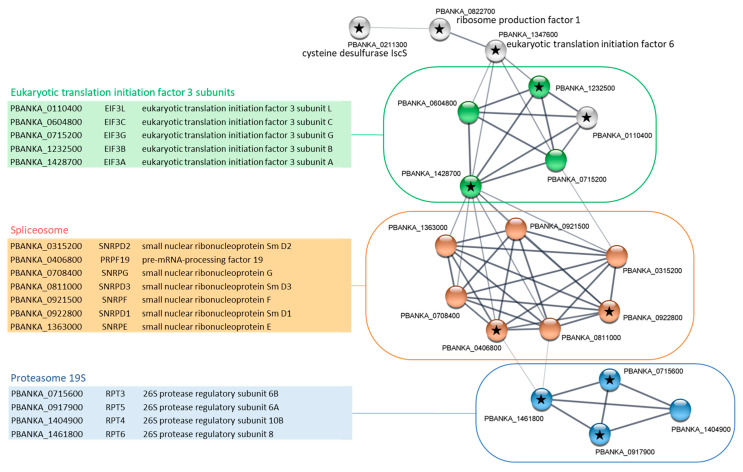
STRING network visualization of PbPP1c-interacting proteins belonging to “cellular component assembly” GO Slim in schizont stages. Using the Cytoscape STRING application, a network was retrieved for the 20 proteins from the cellular component assembly GO Slim of the PbPP1c interactome in schizont stages. With a protein–protein interaction enrichment *p*-value of 4.48 × 10^−10^, the resulting network contained 53 functional associations among 19 proteins; one protein (PBANKA_126600, tubulin gamma chain, putative) with no associations to other proteins in the network was removed. Proteins from the KEGG pathways map03050 (Proteasome), map03013 (RNA transport), and map03040 (Spliceosome) are colored in blue, green, and orange, respectively. Assignation to Eukaryotic translation initiation 3 subunits was conducted manually based on the protein description in PlasmoDB. Black stars indicate proteins with RVxF motifs based on the Wakula consensus sequence [19]. Proteins in each network are listed in adjacent tables showing the PlasmoDB geneID, short name, and protein description. STRING analysis was performed using the 11.0b version of the software.

Next, the proteins involved in each main GO Slim pathways underwent the retrieval of networks for interacting proteins using STRING software (v.11.0b) combined with Cytoscape visualization (v.3.8.2, accessed in July 2021). In the “cellular component assembly” pathway, the results presented in Figure 3 showed a protein–protein interaction network formed of 19 nodes and 53 edges. Three KEGG pathways were significantly enriched in this network (Figure 3). This includes proteasome and spliceosome complexes, confirming the previous analysis [14] and suggesting that these proteins may at least constitute PP1 substrates. In fact, it has been shown that PP1c was able to inhibit human proteasome activities in vitro [24]. In the case of spliceosomes, PP1c has been shown to be an essential player in splicing by contributing to the dephosphorylation of some spliceosome factors [25]. Interestingly, the analysis of data from our study on the PbPP1c interactome shows some overlap with a recent study examining global protein complexes in schizonts from *P. falciparum*, *P. knowlesi*, and *P. berghei* through native-PAGE fractionation coupled with spectrometry and machine learning [26]. This study allowed the identification of >20,000 putative protein–protein interactions, organized in 600 clusters. The reanalysis of these data revealed the presence of 92 putative PP1c interactors (https://plasmogem.shinyapps.io/schizont_interactions/ accessed on 15 September 2021) and GO enrichment of cellular process (Bonferroni adjusted *p*-value = 0.0001), including up to 12 proteins involved in RNA splicing (Bonferroni adjusted *p*-value = 0.009), supportive of our IP-MS of *P. berghei* PP1c and the idea of its role in the regulation of the activity of spliceosomal factors.

In our study, the “cellular component assembly” functional network also included five eukaryotic translation initiation factor 3 (eIF3) subunits, of which four are members of the “RNA transport” KEGG pathway (Figure 3). One of the major nodes of this network was the eIF3 subunit A (eIF3a, PBANKA_1428700) with 11 edges that could be expected to contribute to forming the translation initiation complex. Of note, eIF3a contains four potential RVxF binding motifs, suggesting a potential interaction with PP1c and a regulation of the complex formation by this phosphatase. From our GO Slim analysis, it could also be suggested that PP1c may contribute to the control of the translation process itself (Table 1) in *Plasmodium*. Indeed, 57 proteins belonging to this GO Slim pathway were significantly retrieved in the PbPP1c interactome. This includes the five eIF3 subunits already observed in the “cellular component assembly” network but also 49 ribosomal proteins. STRING analysis of this pathway showed a very high degree of interaction among these proteins (>1400 associations, not shown). Interestingly, phosphorylation is known to be a central mechanism for translational control in mammals, and a role of PP1c in the dephosphorylation of eiF2α to restore translational process upon cell stress has been reported [27]. In addition, eiF2β, which was found in the PP1c interactome in this study but at the limit of significance and also in a recent report [15], was confirmed as a direct interactor of PP1c both in mammals and in *Plasmodium* [28,29].

**Figure 4:**
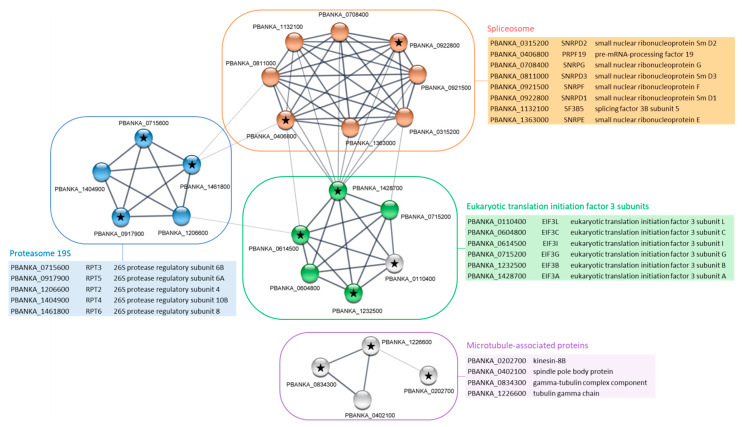
STRING network visualization of PbPP1c-interacting proteins belonging to “cellular component assembly” GO Slim in gametocyte stages. Using the Cytoscape STRING application, a network was retrieved for the 27 proteins from the cellular component assembly GO Slim of the PbPP1c interactome in gametocyte stages. With a protein–protein interaction enrichment *p*-value of 9.99 × 10^−16^, the resulting network contained 70 functional associations among 23 proteins; four proteins (i.e., PBANKA_0211300, cysteine desulfurase IscS; PBANKA_0607800, cytosolic iron–sulfur protein assembly protein 1; PBANKA_0942300, radial spoke head protein; PBANKA_0917400, armadillo repeat protein PF16) with no associations to other proteins in the network were removed. Proteins from KEGG pathways map03050 (proteasome), map03013 (RNA transport), and map03040 (spliceosome) are colored in blue, green, and orange, respectively. Assignation to Eukaryotic translation initiation 3 subunits and to microtubule-associated proteins was conducted manually, based on protein description in PlasmoDB and literature. Black stars indicate proteins with RVxF motifs based on the Wakula consensus sequence [19]. Proteins of each network are listed in adjacent tables showing PlasmoDB geneID, short name, and protein description. STRING analysis was performed using the 11.0b version of the software.

In gametocytes, the GO analysis of PbPP1c-interacting proteins identified 28 pathways as significantly enriched (Supplementary Materials Table S2). Pathways with the highest fold enrichment included spliceosomal SnRNP assembly, microtubule-based movement, microtubule-based processes, movement of cell and DNA replication. When annotations from the full GO terms were converted to GO Slim, PbPP1c was found to cluster with two main pathways (Table 2), including the “cellular component assembly” pathway, which is also observed in schizonts. STRING analysis of the 27 proteins of this pathway showed an additional network in gametocytes, when compared with schizont stages, corresponding to microtubule-associated proteins (Figure 4). Four proteins with no associations with other proteins of the network were removed from the Cytoscape visualization. They included the radial spoke head protein (PBANKA_0942300) and armadillo repeat protein PF16 (PBANKA_0917400): two proteins that are associated with the axoneme and flagella. Of note, the PbPP1c interactome in gametocytes also included kinesin 13 and kinesin 15, together with 14 putative dynein chains, confirming the overall enrichment in microtubule-based processes (Supplementary Materials Table S1). These data are supported by a recent report showing a cyclic accumulation of PbPP1c at the kinetochore during gametogony, suggesting a role for PP1 in regulating mitotic entry and exit [15]. Moreover, several microtubule proteins known to be associated with the spindle or axoneme, some of which were also found in our study, were pulled down with PP1c in gametocytes.

**Figure 5:**
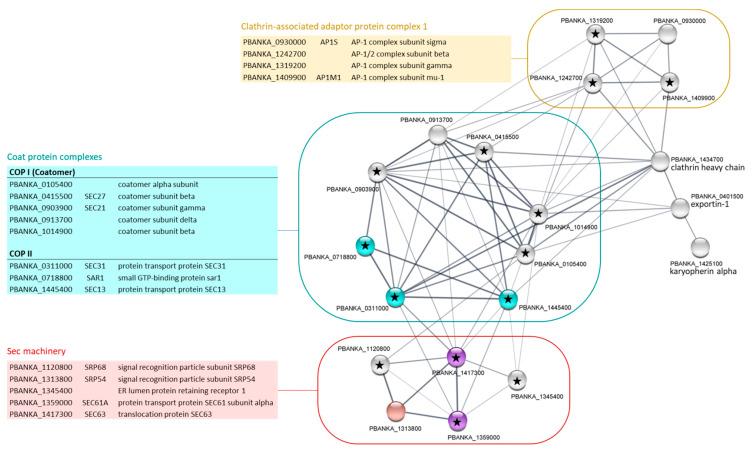
STRING network visualization of PbPP1c-interacting proteins belonging to “protein transport” GO Slim in gametocyte stages. Using the Cytoscape STRING application, a network was retrieved for the 24 proteins from the protein transport GO Slim of the PbPP1c interactome in gametocyte stages. With a protein–protein interaction enrichment *p*-value < 1 × 10^−16^, the resulting network contained 69 functional associations among 20 proteins; four proteins (i.e., PBANKA_0912200, signal recognition particle subunit SRP72; PBANKA_0931200, heat shock protein 101; PBANKA_1217800, signal peptidase complex subunit 2; PBANKA_1359100, nuclear import protein MOG1) with no associations to other proteins in the network were removed. Proteins from KEGG pathways map03060 (protein export) and map04141 (protein processing in endoplasmic reticulum) are colored in blue and red, respectively. Proteins from both of these KEGG pathways are colored in purple. Assignation to coat protein complexes, clathrin-associated adaptor protein complex 1 and to Sec machinery was conducted manually based on protein description in the PlasmoDB and literature. Black stars indicate proteins with RVxF motifs based on the Wakula consensus sequence [19]. Proteins of each network are listed in adjacent tables showing PlasmoDB geneID, short name, and protein description. STRING analysis was performed using the 11.0b version of the database.

The other GO Slim pathway specifically enriched in the PbPP1c interactome in gametocytes was related to protein transport and encompassed 24 proteins (Table 2). The constructed functional protein association network showed 20 nodes and 69 edges, which is a strong enrichment in terms of functional associations when compared to a random network (Figure 5). Few of these proteins belong to significantly enriched KEGG pathways. However, manual inspection based both on PlasmoDB protein description and the literature allowed the identification of three highly interconnected sub-complexes: Clathrin-associated adaptor protein complex (AP-1 complex), coat protein complexes (COPI or coatomer, and COPII), and Sec machinery (or translocase). AP-1 plays a role in protein sorting in the late-Golgi/trans-Golgi network and/or endosomes. It mediates both the recruitment of clathrin to membranes and the recognition of sorting signals within the cytosolic tails of transmembrane cargo molecules. In *Plasmodium*, it has been suggested that AP-1, and especially its µ1 subunit, was involved in rhoptry protein trafficking [30]. Coat protein complexes are non clathrin-based complexes involved in the transport of proteins and lipids destined for the plasma membrane, lysosomes or other post-Golgi compartments (COPII) or in retrograde transport from the Golgi to the endoplasmic reticulum (COPI). In *Plasmodium*, PfSarp1 and PfSec31 (COPII proteins) have been shown to be exported to the erythrocyte cytosol where they appear to play a role in the trafficking of proteins across the erythrocyte cytoplasm [31,32], while PfCOPδ (COPI subunit) was located entirely within the parasite associated with the endoplasmic reticulum [33]. Interestingly, the phosphorylation of COPI β and δ subunits have been shown to be involved in coatomer assembly and function [34,35]. In the gametocyte PbPP1c interactome, five COPs were detected of which four contain at least one potential RVxF binding motif to PP1c. These data, along with the information from PlasmoDB about the phosphorylation status of their counterparts in *P. falciparum*, strongly suggest the contribution of PP1 to the regulation of the function of the COP complex in gametocytes via dephosphorylation.

### 2.4. Proteomic Identification of PbI2 Interactions in Schizonts and Gametocytes

Next, the interactions of the well-known *Plasmodium* Inhibitor-2 of PP1c [12] were investigated, as it appears as one of the proteins with the most significant interaction in schizonts and gametocytes presented in the above data. To this end, the *P. berghei* strain expressing mCherry-I2, described above, was used for IP experiments with anti-RFP antibody. Five biological replicates from the mCherry–PbI2 strain and parental WT were included (Figure 1). Using LC-MS/MS analysis and applying statistical filters, 49 and 35 proteins in schizonts and gametocytes were identified, respectively (Supplementary Materials Table S1, sheets 5 and 6) among which eight proteins were detected in both stages. Of these eight proteins, the bait protein mCherry–PbI2 as well as PbPP1c were found to be highly enriched, confirming the data obtained with the bait mCherry–PbPP1c. The combined PbI2-interacting proteins were first analyzed for GO enrichment annotations. This analysis did not show significant enrichment of any specific pathway in terms of biological processes and molecular processes (data not shown). However, the analysis revealed significant enrichment of cellular components in schizonts (GO term “cytoplasmic microtubule”) and gametocytes (GO term “cytosol”), including two and six proteins respectively (data not shown). Next, in order to set-up possible functional associations, all PbI2-interacting proteins detected in each parasite stage were further searched using the STRING database for interaction mapping. These analyses did not lead to any protein–protein interaction enrichment (*p*-value = 0.237 for schizonts and 0.604 for gametocytes). However, significant functional enrichment in axonemal dynein light chains kinesin motor, tubulin, and actin clusters were observed in the PbI2 interactome in schizonts (data not shown). These clusters involved five proteins: PBANKA_0202700 (kinesin 8B), PBANKA_0925400 (dynein heavy-chain, putative), PBANKA_1018300 (dynein intermediate-chain, putative), PBANKA_0708100 (dynein light-chain 1), and PBANKA_0417700 (alpha tubulin 1). From these data, it appears that PbI2’s functions diverge from those deduced from the human I2 interactome that exhibits a main enriched pathway for the regulation of glycogen biosynthesis (http://thebiogrid.org/ID5504, accessed on 15 September 2021).

### 2.5. Identification of Shared Proteins and Interactions of PP1c and I2 in Schizonts and Gametocytes

**Figure 6:**
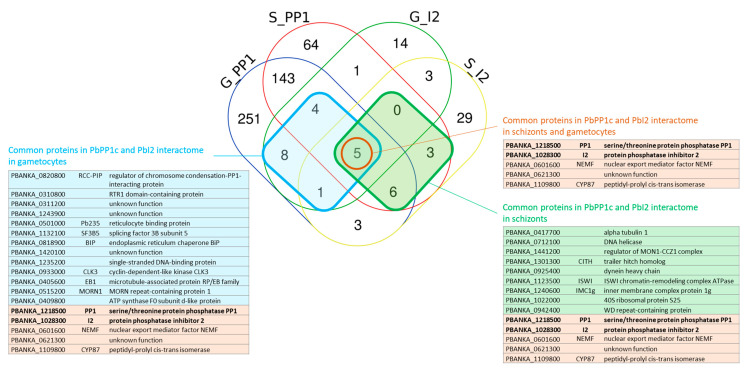
Distribution analysis of the PbPP1c and PbI2 potential partners identified in IP/MS in *P. berghei* schizonts and gametocytes. The Venn diagram shows the number of PbPP1c and PbI2 partners identified in schizonts (S_PP1 and S_I2, respectively) and gametocytes (G_PP1 and G_I2, respectively). Common proteins in the PbPP1c and PbI2 interactomes in schizonts or in gametocytes are boxed in green and blue squares, respectively, and listed in adjacent tables showing the PlasmoDB geneID, short name, and protein description with the same color code. Common proteins in PbPP1c and PbI2 interactomes in schizonts and gametocytes are circled and listed in orange. This diagram was created using the Venn online tool https://bioinformatics.psb.ugent.be/webtools/Venn/ accessed on 4 May 2021.

The assembly of PP1c with a given regulator is expected to select appropriate substrates to target and/or to regulate the dephosphorylation process by controlling access to the catalytic site. It has been shown that *Plasmodium* PP1c interacts not only with I2 in vitro but also in the parasite by IP of PP1c and reciprocal IP of I2 (this study) [12]. We therefore searched for possible common proteins among the two interactomes. This allowed the identification of 14 and 18 common proteins in schizonts and gametocytes, respectively (I2 and PP1c included) (Figure 6). GO enrichment and STRING analyses of these sets of common proteins did not reveal significant scores which could be due to the low number of shared proteins. Further analysis of shared proteins allowed us to identify five common proteins regardless of the parasite stage examined (Figure 6). This included PP1, I2, nuclear export mediator factor (NEMF), peptidyl-prolyl cis-trans isomerase, and PBANKA_0621300 with unknown function, designating these proteins as potential substrates for the regulation of their phosphorylation status by the PP1–I2 holoenzyme. The detection of these common proteins in PP1c and I2 interactomes is likely specific and independent of the mCherry tag, as these proteins were not detected in the interactome of mCherry–PbGEXP15 in schizonts and gametocytes that we recently reported as a partner of PP1c [14]. When the common partners were examined according to the parasite stage (schizonts versus gametocytes), our findings are supportive of the capacity of the I2/PP1c complex to form distinct co-complexes. Moreover, they suggest that the proteins of these co-complexes could not only be potential substrates but also play the role of transporters and/or adaptors of the I2/PP1c complex. Of note, among the common proteins of both interactomes in gametocytes, a direct and specific partner of PP1c that we previously reported and designated RCC–PIP was detected [18]. This partner that binds PP1c via its RVxF binding motif has also been shown to interact with CDPK7 kinase through its RCC domain, suggesting a subtle control of phosphorylation levels of partners to accurately coordinate the binding of the PP1c.

### 2.6. Conclusions

In this study, PP1c and I2 interactomes showed a plethora of interactions uncovering important structural and regulatory networks that seem to be dynamic with respect to the stage of *Plasmodium*. Our study of the PP1c interactome confirmed the presence of seven direct partners in the complex that we have previously shown using recombinant proteins. Six partners out of seven exhibited an RVxF motif that has been shown as the main contributor to the binding to PP1c. We report the presence of an additional 296 proteins with at least one consensus RVxF-binding motif, suggesting their capacity to bind PP1c. Further, the potential role of PP1c in common pathways present in schizonts and gametocytes and its interactome in the latter revealed, for the first time, the enrichment of a specific pathway involved in protein transport. These diverse pathways can constitute platforms for better understanding the role of partners on PP1c functions and vice versa to gain new insights on the plasticity and functional repurposing of this phosphatase. Overall, *Plasmodium* PP1c holoenzymes justify further investigations for drug targeting by including not only specific essential partners and related pathways but also those with human orthologs which may have specific features that allow for the selective inhibition of the conserved holoenzymes.

## 3. Materials and Methods

### 3.1. Ethics Statement

All animal experiments were carried out in accordance with the French National Guidelines. Protocols and procedures on mice were supervised and approved by the CEEA-75 Comité d’Ethique en Expérimentation Animale, Nord-Pas de Calais, France: APAFIS#18905-2019020111166978v2. Date of approval 29 July 2019.

Mice used were male CD1 (22–24 g) from Charles River Laboratories maintained in filtered cages in an Animal Biosafety Level 2 facility at the Institut Pasteur de Lille.

### 3.2. Materials

The *Plasmodium berghei* strain used in this study was a PbGFP ANKA line kindly provided by O. Silvie (Université Pierre et Marie Curie, Paris, France). Plasmid pL1886 was given by B. Franke-Fayard (Leiden University Medical Center, Leiden, The Netherlands). The primers used are described in Supplementary Materials Table S3.

### 3.3. Generation of P. berghei Transgenic Parasites

The PbPP1c–mCherry line was obtained by single homologous recombination as previously described [14]. Similarly, a C-terminal mCherry-tagged PbI2 was generated by single homologous recombination of a 1436 pb region of PbI2 without the stop codon (Pr3–Pr2, Supplementary Materials Table S3 and Figure S1A) inserted into the pL1886 vector and NdeI-linearized before transfection into the PbGFP ANKA strain.

Genotyping was performed on total DNA from parental and transfected parasites extracted from schizont pellets using the KAPA Express Extract Kit (KAPA BioSystem Inc, Wilmington, MA, USA). Transfected parasites were genotyped by diagnostic PCR using primers indicated in the Supplementary Materials Figure S1A and Table S3: Pr1–Pr2 for wild locus detection and Pr1–Pr4 for 3’ integration.

### 3.4. Isolation of P. berghei Schizonts and Gametocytes

Parasite stages were isolated as previously described [14]. Briefly, to obtain schizonts, blood from infected mice (parasitemia around 5%) was collected by intracardiac puncture and incubated 20 h at 37 °C, 54 rpm in schizont media (RPMI 1640 medium containing 25 mM HEPES, 0.4% Albumax, 0.2 mM hypoxanthine, and 20 µg/mL gentamycin). Then, schizonts were purified on a 60% nycodenz gradient (27.6% *w*/*v* Nycodenz in 5 mM Tris-HCl, 3 mM KCl, 0.3 mM EDTA, and pH 7.2) for 20 min at 450 g.

To purify gametocytes, mice were treated with phenylhydrazine (200 μL, 6 mg/mL, Sigma–Aldrich, St. Louis, MO, USA, by intraperitoneal route) 3 days before the infection. At day 3 post-infection (p.i.), mice were treated with sulfadiazine for 2 days (20 mg/L in drinking water, Sigma–Aldrich, St. Louis, MO, USA). Two days later, blood was collected by cardiac puncture and gametocytes were purified on a 48% nycodenz gradient in coelenterazine loading buffer (CLB: PBS, 20 mM HEPES, 20 mM glucose, 4 mM sodium bicarbonate, 1 mM EDTA, 0.1% *w*/*v* bovine serum albumin, and pH 7.25) for 10 min at 1082 g.

### 3.5. Immunoblot Assays

Immunoblot of PbI2–mCherry was carried out on schizont stages obtained as described above. Parental parasites were used as a control. Pellets of schizonts were directly resuspended in Laemmli buffer. The presence of PbI2–mCherry protein was observed by Western blot with anti-RFP (red fluorescent protein) pAb (1:2000, MBL, Woburn, MA, USA, Cat#PM005) followed by goat anti-rabbit IgG-HRP (1:20,000, Sigma–Aldrich, St. Louis, MO, USA).

### 3.6. Sample Preparation and Immunoprecipitation

*P. berghei* schizonts or gametocytes of PbPP1c–mCherry, PbI2–mCherry, or parental wild-type strain (control) were purified as described above.

Immunoprecipitation experiments were performed using 5 biological replicates of each strain. Each biological replicate contained 6 isolated rings of schizonts or gametocytes, each purified from one infected mouse. Soluble proteins extractions and immunoprecipitation assays were performed as previously described [14]. Briefly, purified schizonts or gametocytes were suspended in lysis buffer containing 50 mM Tris, 0.5% Triton X100, and protease inhibitor cocktail (Roche, Basel, Switzerland) at pH 8. After 10 freeze–thaw cycles and sonication, soluble fractions were obtained after repeated centrifugations (5 h, 13,000 rpm, 4 °C). These soluble fractions were incubated with RFP-Trap1_A beads (Chromotek, Planegg, Germany) overnight at 4 °C on a rotating wheel in dilution buffer containing 20 mM Tris, 150 mM NaCl, 0.5% Triton X-100, and protease inhibitor cocktail (Roche, Basel, Switzerland) at pH 7.5. After centrifugation (2500× *g* for 2 min), the beads were washed 10 times with dilution buffer and elution was performed in Laemmli buffer.

### 3.7. Sample Preparation for Interactome Analysis

Immunoprecipitation eluates were supplemented with SDS to a final concentration of 5% for protein digestion. S-Trap^TM^ micro spin column (Protifi, Farmingdale, NY, USA) digestion was performed on immunoprecipitation eluates according to manufacturer’s protocol but using two extra washing steps for thorough SDS elimination. Samples were digested with 2 µg of trypsin (Promega, Madison, WI, USA) at 47 °C for 2 h. After elution, peptides were vacuum dried.

### 3.8. NanoLC-MS/MS Protein Identification and Quantification

Samples were resuspended in 35 µL of 10% ACN and 0.1% TFA in HPLC-grade water. For immunoprecipitation experiments, single-shot MS analysis was performed. For each run, 2 µL was injected in a nanoRSLC-Q Exactive PLUS (RSLC Ultimate 3000) (Thermo Scientific, Waltham MA, USA). Peptides were loaded onto a µ-precolumn (Acclaim PepMap 100 C18, cartridge, 300 µm i.d. × 5 mm, 5 µm) (Thermo Scientific, Waltham MA, USA), and were separated on a 50 cm reversed-phase liquid chromatographic column (0.075 mm ID, Acclaim PepMap 100, C18, 2 µm) (Thermo Scientific, Waltham MA, USA). Chromatography solvents were (A) 0.1% formic acid in water and (B) 80% acetonitrile and 0.08% formic acid. Peptides were eluted from the column with the following gradient 5–40% B (120 min) and 40–80% (1 min). At 121 min, the gradient stayed at 80% for 5 min, and at 126 min, it returned to 5% to re-equilibrate the column for 20 min before the next injection. One blank was run between each replicate to prevent sample carryover. Peptides eluting from the column were analyzed by data dependent MS/MS, using the top-10 acquisition method and fragmented using higher-energy collisional dissociation (HCD). Briefly, the instrument settings were as follows: the resolution was set to 70,000 for MS scans and 17,500 for the data dependent MS/MS scans in order to increase speed. The MS AGC target was set to 3 × 10^6^ counts with a maximum injection time set to 60 ms, while the MS/MS AGC target was set to 1 × 10^5^ with maximum injection time set to 120 ms. The MS scan range was from 400 to 2000 *m*/*z*. Dynamic exclusion was set to a 30 s duration.

The MS files were searched with the MaxQuant software version 1.6.6.0 and searched with the Andromeda search engine against the database of Mus musculus from Swiss-Prot 07/2020 and *Plasmodium berghei* ANKA from PlasmoDB (v.37). To search parent mass and fragment ions, we set a mass deviation of 3 and 20 ppm, respectively. The minimum peptide length was set to 7 amino acids and strict specificity for trypsin cleavage was required, allowing up to two missed cleavage sites. Carbamidomethylation (Cys) was set as fixed modification, whereas oxidation (Met) and N-term acetylation were set as variable modifications. The false discovery rates (FDRs) at the protein and peptide levels were set to 1%. Scores were calculated in MaxQuant as described previously [36]. The reverse and common contaminants hits were removed from MaxQuant output. Proteins were quantified according to the MaxQuant label-free algorithm using LFQ intensities; protein quantification was obtained using at least 2 peptides per protein. Matches between runs were allowed.

Statistical and bioinformatic analyses, were performed with Perseus software (version 1.6.12.0) freely available at www.perseus-framework.org [37]. For statistical comparison, we set six groups: PbWT schizonts and gametocytes as the negative control (S_WT, G_WT), immunoprecipitates from schizonts and gametocytes expressing PbPP1c–mCherry (i.e., S_PP1 and G-PP1), and immunoprecipitates from schizonts and gametocytes expressing PbI2–mCherry (i.e., S_I2 and G_I2). Each group contained five biological replicates. We then filtered the data to keep only proteins with at least 5 valid values in at least one group. Next, the data were imputed to fill missing data points by creating a Gaussian distribution of random numbers with a standard deviation of 33% relative to the standard deviation of the measured values and 1.8 standard deviation downshift of the mean to simulate the distribution of low signal values. We performed a *t*-test represented on a volcano plot, FDR < 0.05, S0 = 1.

### 3.9. Proteome Analysis

The RVxF binding motifs (consensus sequence (R/K)-X0-1(V/I)-{P}-(F/W), where X can be any amino acid and {P} any residue except proline [19]) were identified using the tool “Protein Motif Pattern” available on the PlasmoDB website.

The raw spectral data from tagged baits and wild-type experiments were used to interrogate the *Plasmodium* proteome.

The GO (gene ontology) or GO slim enrichment analyses were conducted on the PlasmoDB website with the following parameters: ontology = biological process; evidence: computed and curated; limit to GO slim terms: no for global and yes for GO slim; *p*-value cutoff = 0.05. For statistical relevance we kept the GO enrichment showing a Bonferroni adjusted *p*-value ≤ 0.05.

The different protein subnetworks were based on the results from STRING database (Search Tool for the Retrieval of Interacting Genes/Proteins v.11.0b; https://string-db.org/; [38]) and visualized on Cytoscape (https://cytoscape.org/, v.3.8.2, accessed on 1 July 2021).

The Venn diagram was established with the Venn online tool (https://bioinformatics.psb.ugent.be/webtools/Venn/ accessed on 4 May 2021).

The mass spectrometry proteomics data were deposited with the ProteomeXchange Consortium via the PRIDE [39] partner repository with the data set identifier: PXD029283.

## Figures and Tables

**Table 1:** Analysis of biological processes enrichment in the PbPP1c interactome in schizonts limited to GO SLIM terms.

GO ID	GO Term	Number of Genes Detected ^1^	Fold Enrichment	*p*-Value	Benjamini FDR	Bonferroni Adjusted *p*-Value
GO:0022607	Cellular component assembly	20/108	2.6	4.63 × 10^−5^	2.55 × 10^−4^	1.53 × 10^−3^
GO:0065003	Protein-containing complex assembly	18/80	3.16	6.93 × 10^−6^	4.57 × 10^−5^	2.29 × 10^−4^
GO:0022618	Ribonucleoprotein complex assembly	13/43	4.25	4.39 × 10^−6^	3.62 × 10^−5^	1.45 × 10^−4^
GO:0009058	Biosynthetic process	71/531	1.88	4.53 × 10^−9^	7.47 × 10^−8^	1.49 × 10^−7^
GO:0034641	Cellular nitrogen compound metabolic process	87/756	1.62	7.49 × 10^−8^	8.24 × 10^−7^	2.47 × 10^−6^
GO:0006412	Translation	57/232	3.45	4.86 × 10^−19^	1.60 × 10^−17^	1.60 × 10^−17^

^1^ Number of genes detected/total number of genes in the background with this term.

**Table 2:** Analysis of biological processes enrichment in the PbPP1c interactome in gametocytes limited to GO SLIM terms.

GO ID	GO Term	Number of Genes Detected ^1^	Fold Enrichment	*p*-Value	Benjamini FDR	Bonferroni Adjusted *p*-Value
GO:0022607	Cellular component assembly	27/108	2.18	4.48 × 10^−5^	1.04 × 10^−3^	1.88 × 10^−3^
GO:0065003	Protein-containing complex assembly	22/80	2.4	4.97 × 10^−5^	1.04 × 10^−3^	2.09 × 10^−3^
GO:0022618	Ribonucleoprotein complex assembly	14/43	2.84	1.75 × 10^−4^	2.45 × 10^−3^	7.35 × 10^−3^
GO:0015031	Protein transport	24/109	1.92	9.32 × 10^−4^	9.79 × 10^−3^	3.91 × 10^−2^

^1^ Number of genes detected/total number of genes in the background with this term.

## Data Availability

The mass spectrometry proteomics data were deposited with the Proteo-meXchange Consortium via the PRIDE partner repository (https://www.ebi.ac.uk/pride/) with the data set identifier: PXD029283.

## Supplementary Materials

The following are available online at https://www.mdpi.com/article/10.3390/ijms23031069/s1.

## References

[1] Menard D., Dondorp A. (2017). Antimalarial Drug Resistance: A Threat to Malaria Elimination. Cold Spring Harb. Perspect. Med..

[2] Casamayor A., Ariño J. (2020). Controlling Ser/Thr protein phosphatase PP1 activity and function through interaction with regulatory subunits. Adv. Protein Chem. Struct. Biol..

[3] Barker H.M., Brewis N.D., Street A.J., Spurr N.K., Cohen P.T.W. (1994). Three genes for protein phosphatase 1 map to different human chromosomes: Sequence, expression and gene localisation of protein serine/threonine phosphatase 1 beta (PPP1CB). Biochim. Biophys. Acta.

[4] Bhattacharyya M.K., Hong Z., Kongkasuriyachai D., Kumar N. (2002). *Plasmodium falciparum* protein phosphatase type 1 functionally complements a glc7 mutant in *Saccharomyces cerevisiae*. Int. J. Parasitol..

[5] Yokoyama D., Saito-Ito A., Asao N., Tanabe K., Yamamoto M., Matsumura T. (1998). Modulation of the growth of *Plasmodium falciparum* in vitro by protein serine/threonine phosphatase inhibitors. Biochem. Biophys. Res. Commun..

[6] Paul A.S., Miliu A., Paulo J.A., Goldberg J.M., Bonilla A.M., Berry L., Séveno M., Braun-Breton C., Kosber A.L., Elsworth B. (2020). Co-option of *Plasmodium falciparum* PP1 for egress from host erythrocytes. Nat. Commun..

[7] Choy M.S., Hieke M., Kumar G.S., Lewis G.R., Gonzalez-DeWhitt K.R., Kessler R.P., Stein B.J., Hessenberger M., Nairn A.C., Peti W. (2014). Understanding the antagonism of retinoblastoma protein dephosphorylation by PNUTS provides insights into the PP1 regulatory code. Proc. Natl. Acad. Sci. USA.

[8] Hendrickx A., Beullens M., Ceulemans H., Den Abt T., Van Eynde A., Nicolaescu E., Lesage B., Bollen M. (2009). Docking motif-guided mapping of the interactome of protein phosphatase-1. Chem. Biol..

[9] Heroes E., Lesage B., Gornemann J., Beullens M., Van Meervelt L., Bollen M. (2013). The PP1 binding code: A molecular-lego strategy that governs specificity. FEBS J..

[10] Moorhead G.B.G., Trinkle-Mulcahy L., Nimick M., De Wever V., Campbell D.G., Gourlay R., Lam Y.W., Lamond A.I. (2008). Displacement affinity chromatography of protein phosphatase one (PP1) complexes. BMC Biochem..

[11] Hollin T., De Witte C., Lenne A., Pierrot C., Khalife J. (2016). Analysis of the interactome of the Ser/Thr Protein Phosphatase type 1 in *Plasmodium falciparum*. BMC Genom..

[12] Freville A., Cailliau-Maggio K., Pierrot C., Tellier G., Kalamou H., Lafitte S., Martoriati A., Pierce R.J., Bodart J.F., Khalife J. (2013). *Plasmodium falciparum* encodes a conserved active inhibitor-2 for Protein Phosphatase type 1: Perspectives for novel anti-plasmodial therapy. BMC Biol..

[13] Pierrot C., Zhang X., Zhangi G., Freville A., Rebollo A., Khalife J. (2018). Peptides derived from *Plasmodium falciparum* leucine-rich repeat 1 bind to serine/threonine phosphatase type 1 and inhibit parasite growth in vitro. Drug Des. Dev. Ther..

[14] Hollin T., De Witte C., Freville A., Guerrera I.C., Chhuon C., Saliou J.M., Herbert F., Pierrot C., Khalife J. (2019). Essential role of GEXP15, a specific Protein Phosphatase type 1 partner, in *Plasmodium berghei* in asexual erythrocytic proliferation and transmission. PLoS Pathog..

[15] Zeeshan M., Pandey R., Subudhi A.K., Ferguson D.J.P., Kaur G., Rashpa R., Nugmanova R., Brady D., Bottrill A.R., Vaughan S. (2021). Protein phosphatase 1 regulates atypical mitotic and meiotic division in *Plasmodium* sexual stages. Commun. Biol..

[16] Daher W., Browaeys E., Pierrot C., Jouin H., Dive D., Meurice E., Dissous C., Capron M., Tomavo S., Doerig C. (2006). Regulation of protein phosphatase type 1 and cell cycle progression by PfLRR1, a novel leucine-rich repeat protein of the human malaria parasite *Plasmodium falciparum*. Mol. Microbiol..

[17] Freville A., Landrieu I., Garcia-Gimeno M.A., Vicogne J., Montbarbon M., Bertin B., Verger A., Kalamou H., Sanz P., Werkmeister E. (2012). *Plasmodium falciparum* inhibitor-3 homolog increases protein phosphatase type 1 activity and is essential for parasitic survival. J. Biol. Chem..

[18] Lenne A., De Witte C., Tellier G., Hollin T., Aliouat E.M., Martoriati A., Cailliau K., Saliou J.-M., Khalife J., Pierrot C. (2018). Characterization of a Protein Phosphatase Type-1 and a Kinase Anchoring Protein in *Plasmodium falciparum*. Front. Microbiol..

[19] Wakula P., Beullens M., Ceulemans H., Stalmans W., Bollen M. (2003). Degeneracy and function of the ubiquitous RVXF motif that mediates binding to protein phosphatase-1. J. Biol. Chem..

[20] Beullens M., Stalmans W., Bollen M. (1996). Characterization of a Ribosomal Inhibitory Polypeptide of Protein Phosphatase-1 from Rat Liver. Eur. J. Biochem. FEBS.

[21] Hirano K., Ito M., Hartshorne D.J. (1995). Interaction of the Ribosomal Protein, L5, with Protein Phosphatase Type 1. J. Biol. Chem..

[22] Hutchinson J.A., Shanware N.P., Chang H., Tibbetts R.S. (2011). Regulation of ribosomal protein S6 phosphorylation by casein kinase 1 and protein phosphatase 1. J. Biol. Chem..

[23] Chamousset D., De Wever V., Moorhead G.B., Chen Y., Boisvert F.-M., Lamond A.I., Trinkle-Mulcahy L. (2010). RRP1B targets PP1 to mammalian cell nucleoli and is associated with Pre-60S ribosomal subunits. Mol. Biol. Cell.

[24] Zhang F., Hu Y., Huang P., Toleman C.A., Paterson A.J., Kudlow J.E. (2007). Proteasome Function Is Regulated by Cyclic AMP-dependent Protein Kinase through Phosphorylation of Rpt6. J. Biol. Chem..

[25] Shi Y., Reddy B., Manley J.L. (2006). PP1/PP2A Phosphatases Are Required for the Second Step of Pre-mRNA Splicing and Target Specific snRNP Proteins. Mol. Cell.

[26] Hillier C., Pardo M., Yu L., Bushell E., Sanderson T., Metcalf T., Herd C., Anar B., Rayner J.C., Billker O. (2019). Landscape of the *Plasmodium* Interactome Reveals Both Conserved and Species-Specific Functionality. Cell Rep..

[27] Choy M.S., Yusoff P., Lee I.C., Newton J.C., Goh C.W., Page R., Shenolikar S., Peti W. (2015). Structural and Functional Analysis of the GADD34:PP1 eIF2α Phosphatase. Cell Rep..

[28] Tellier G., Lenne A., Cailliau-Maggio K., Cabezas-Cruz A., Valdes J.J., Martoriati A., Aliouat el M., Gosset P., Delaire B., Freville A. (2016). Identification of *Plasmodium falciparum* Translation Initiation eIF2beta Subunit: Direct Interaction with Protein Phosphatase Type 1. Front. Microbiol..

[29] Wakula P., Beullens M., van Eynde A., Ceulemans H., Stalmans W., Bollen M. (2006). The translation initiation factor eIF2beta is an interactor of protein phosphatase-1. Biochem. J..

[30] Kaderi Kibria K.M., Rawat K., Klinger C.M., Datta G., Panchal M., Singh S., Iyer G.R., Kaur I., Sharma V., Dacks J.B. (2015). A role for adaptor protein complex 1 in protein targeting to rhoptry organelles in *Plasmodium falciparum*. Biochim. Biophys. Acta.

[31] Adisa A., Albano F.R., Reeder J., Foley M., Tilley L. (2001). Evidence for a role for a *Plasmodium falciparum* homologue of Sec31p in the export of proteins to the surface of malaria parasite-infected erythrocytes. J. Cell Sci..

[32] Albano F.R., Berman A., La Greca N., Hibbs A.R., Wickham M., Foley M., Tilley L. (1999). A homologue of Sar1p localises to a novel trafficking pathway in malaria-infected erythrocytes. Eur. J. Cell Biol..

[33] Adisa A., Rug M., Foley M., Tilley L. (2002). Characterisation of a δ-COP homologue in the malaria parasite, *Plasmodium falciparum*. Mol. Biochem. Parasitol..

[34] Luo P.M., Boyce M. (2019). Directing Traffic: Regulation of COPI Transport by Post-translational Modifications. Front. Cell Dev. Biol..

[35] Sheff D., Lowe M., Kreis T.E., Mellman I. (1996). Biochemical Heterogeneity and Phosphorylation of Coatomer Subunits. J. Biol. Chem..

[36] Cox J., Mann M. (2008). MaxQuant enables high peptide identification rates, individualized p.p.b.-range mass accuracies and proteome-wide protein quantification. Nat. Biotechnol..

[37] Tyanova S., Temu T., Sinitcyn P., Carlson A., Hein M.Y., Geiger T., Mann M., Cox J. (2016). The Perseus computational platform for comprehensive analysis of (prote)omics data. Nat. Methods.

[38] Szklarczyk D., Gable A.L., Lyon D., Junge A., Wyder S., Huerta-Cepas J., Simonovic M., Doncheva N.T., Morris J.H., Bork P. (2019). STRING v11: Protein-protein association networks with increased coverage, supporting functional discovery in genome-wide experimental datasets. Nucleic Acids Res..

[39] Perez-Riverol Y., Csordas A., Bai J., Bernal-Llinares M., Hewapathirana S., Kundu D.J., Inuganti A., Griss J., Mayer G., Eisenacher M. (2019). The PRIDE database and related tools and resources in 2019: Improving support for quantification data. Nucleic Acids Res..

