# Supplementary material for: Mapping PP1c and Its Inhibitor 2 Interactomes Reveals Conserved and Specific Networks in Asexual and Sexual Stages of Plasmodium
Source: Int J Mol Sci. 2022 Jan 19;23(3):1069. doi: 10.3390/ijms23031069 (PMC8835298; doi:10.3390/ijms23031069)
Supplement: Supplementary file 1 [file ijms-23-01069-s001.zip › Supplementary FigS1.pdf]

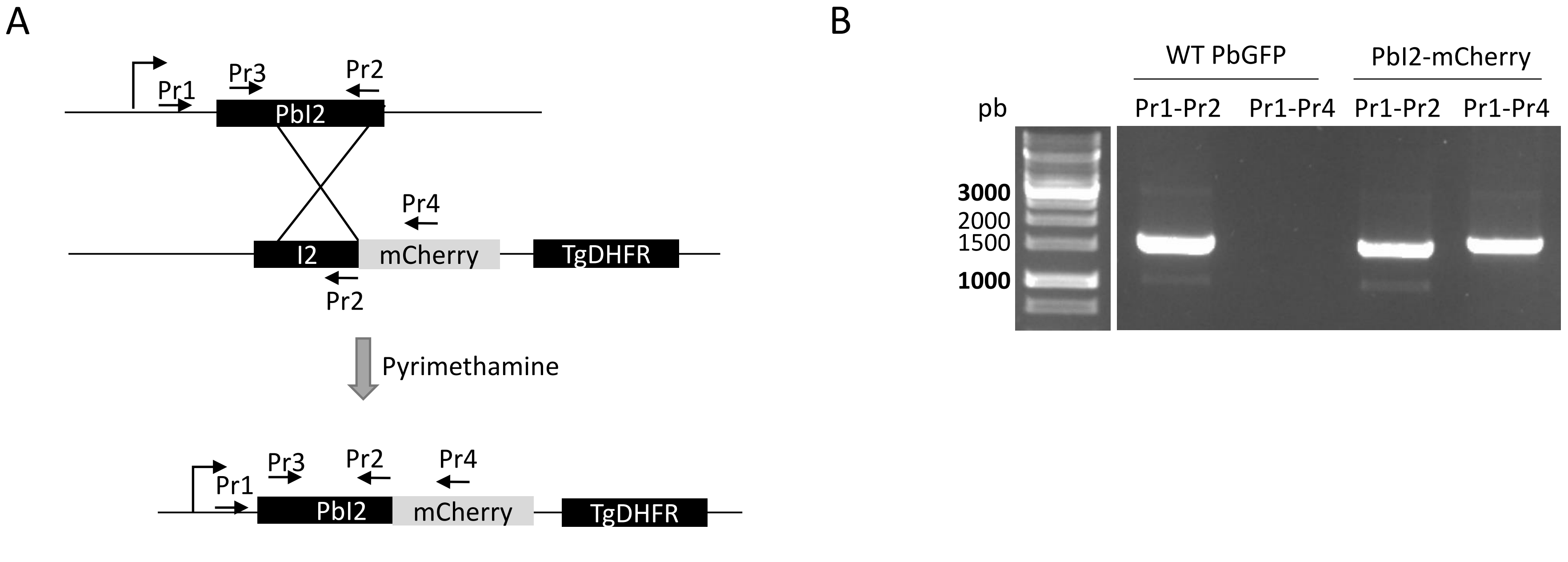

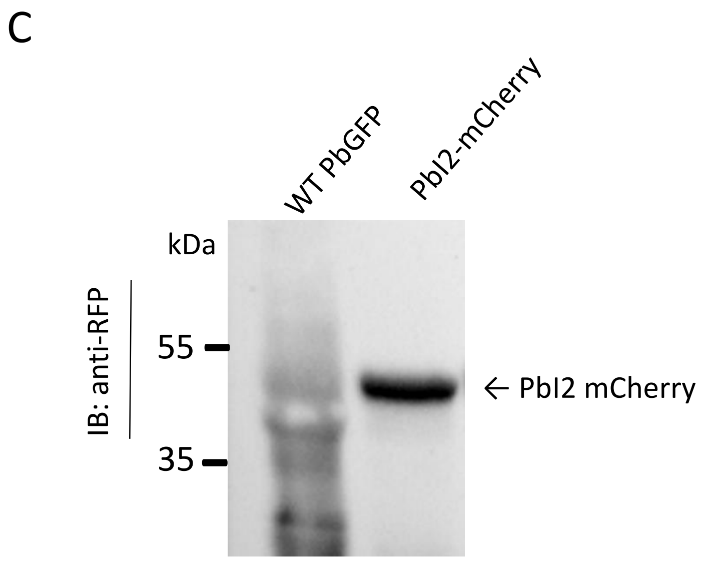

Figure S1: Generation of PbI2-mCherry line.

A) Schematic representation of the C-ter mCherry tagging of PbI2 using single homologous recombination. Are represented the plasmid with the mCherry tag and the TgDHFR selection marker. Transgenic parasites were selected using pyrimethamine. The position of the primers (Supplementary Table S3) used for diagnostic PCR are indicated by arrows.

B) Diagnostic PCR of PbI2-mCherry (lanes 3-4) compared to WT PbGFP parasites (lanes 1-2). Lanes 1 and 3: control PCR: amplification of a portion of PbI2 (Pr1 and Pr2). Lanes 2 and 4 : 5' integration (Pr1-Pr4).

C) Western-blot analysis performed on PbI2-mCherry and WT PbGFP ANKA extracts using a monoclonal anti-RFP antibody. Lane 1 corresponds to WT PbGFP, used as negative control and lane 2 corresponds to PbI2-mCherry at the expected size.
